# Middle managers’ role in implementing evidence-based practices in healthcare: a systematic review

**DOI:** 10.1186/s13012-018-0843-5

**Published:** 2018-12-12

**Authors:** Sarah Birken, Alecia Clary, Amir Alishahi Tabriz, Kea Turner, Rosemary Meza, Alexandra Zizzi, Madeline Larson, Jennifer Walker, Martin Charns

**Affiliations:** 1 Department of Health Policy and Management, Gillings School of Global Public Health, The University of North Carolina at Chapel Hill, 1103E McGavran-Greenberg, 135 Dauer Drive, Campus Box 7411, Chapel Hill, NC 27599-7411 USA; 2 Department of Psychology, University of Washington, Guthrie Hall, Room 119A, UW Box 351525, Seattle, WA 98195 USA; 3 Department of Educational Psychology, University of Minnesota, 104 Burton Hall, 178 Pillsbury Dr. S.E, Minneapolis, MN 55455 USA; 4 The University of North Carolina at Chapel Hill, Health Sciences Library, 335 S. Columbia Street, Campus Box CB 7585, Chapel Hill, NC 27599-7411 USA; 5 Center for Healthcare Organization and Implementation Research (CHOIR), VA Boston Healthcare System, 150 South Huntington Avenue (152M), Jamaica Plain Campus, Building 9, Boston, MA 02130 USA; 6 Department of Health Law, Policy & Management, School of Public Health, Boston University, 715 Albany St. Talbot Building, Boston, MA 02118 USA

**Keywords:** Middle manager, Supervisor, Leader, Implementation, Healthcare, Systematic review, Implementation climate, Evidence-based practice

## Abstract

**Background:**

Middle managers are in a unique position to promote the implementation of evidence-based practices (EBPs) in healthcare organizations, yet knowledge of middle managers’ role in implementation and determinants (e.g., individual-, organizational-, and system-level factors) which influence their role remains fractured, spanning decades and disciplines. To synthesize understanding, we undertook a systematic review of studies of middle managers’ role in healthcare EBP implementation and determinants of that role.

**Methods:**

We searched MEDLINE/PubMed and Business Source Complete (Ebsco) for literature on middle managers’ role in healthcare EBP implementation and its determinants. We abstracted data from records that met inclusion criteria (i.e., written in English, peer-reviewed, and reporting either a protocol or results of an empirical study) into a matrix for analysis. We summarized categorical variables using descriptive statistics. To analyze qualitative data, we used a priori codes and then allowed additional themes to emerge.

**Results:**

One hundred five records, spanning across several countries and healthcare settings and relating to a range of EBPs, met our inclusion criteria. Studies of middle managers’ role in healthcare EBP implementation and its determinants substantially increased from 1996 to 2015. Results from included studies suggest that middle managers shape implementation climate in addition to fulfilling the four roles hypothesized in extant theory of middle managers’ role in implementation. However, extant studies offered little understanding of determinants of middle managers’ role.

**Conclusions:**

Our findings suggest that middle managers may play an important role in facilitating EBP implementation. Included studies offered little understanding regarding the relative importance of various roles, potential moderators of the relationship between middle managers’ roles and EBP implementation, or determinants of middle managers’ role in EBP implementation. Future studies should seek to understand determinants and moderators of middle managers’ role. Clearer understanding may facilitate the translation of evidence into practice.

**Electronic supplementary material:**

The online version of this article (10.1186/s13012-018-0843-5) contains supplementary material, which is available to authorized users.

## Background

Middle managers- employees who supervise frontline employees and are supervised by an organization’s top managers (e.g., project managers, nurse managers, team managers) [1] are in a unique position to promote the implementation of evidence-based practices (EBPs) in healthcare organizations. Interest in middle managers’ role in EBP implementation has increased; several empirical studies have recently been published on the topic [2–4]. In large part, these studies have called to task extant studies, which largely take for granted that middle managers will enact top managers’ directives to implement EBPs [5–7]. For example, Chuang, Jason, and Morgan found that middle managers supported implementation when EBPs met their organization’s needs and when they had control over implementing EBPs [8]. Urquhart and colleagues found that middle managers’ support was critical for the successful implementation of reporting tools in cancer care [2]. Birken and colleagues found that middle managers’ commitment influences implementation effectiveness when middle managers are proactive [3] and when they receive support from top managers [4]. The topic received additional attention upon the publication of a theory of middle managers’ role in healthcare EBP implementation [1]. The theory suggests that middle managers fill structural holes in healthcare organizations, promoting implementation by engaging in four roles: (1) diffusing information (i.e., using media to provide comprehensive and relevant information to stakeholders), (2) synthesizing information (i.e., combining, interpreting and explaining different elements to synthesize a cohesive and comprehensive piece of information), (3) mediating between strategy and day-to-day activities (i.e., addressing concerns raised by frontline employees and enabling them to fulfill their implementation-related responsibilities by overcoming barriers, holding them accountable, and coaching them), and (4) selling EBP implementation (i.e., presenting, convincing, and encouraging stakeholders to participate in implementation). Per this theory, the relationship between these roles and implementation is mediated by implementation climate (i.e., employees’ collective perception of the extent to which EBP implementation is rewarded, supported, or expected in a given organization [9]). Premised on the notion that middle managers may be levers for change in healthcare organizations, the theory intended to stimulate research and promote convergence of understanding regarding middle managers’ role in healthcare EBP implementation.

Despite increased interest in the topic, knowledge of middle managers’ role in healthcare EBP implementation and its determinants remains fractured, spanning decades and disciplines. To synthesize understanding, we present results from a systematic review of the literature on middle managers’ role in healthcare EBP implementation and its determinants. Specifically, our objectives were to (1) summarize the literature on middle managers’ role in implementation, (2) summarize the literature on determinants of middle managers’ role in implementation, and (3) examine the convergence between extant evidence and the roles posited by the theory of middle managers’ role in implementation. The resulting knowledge of extant evidence regarding middle managers’ role produced from this review will help to define an agenda for future research on the topic—what is known and what warrants new research. Additionally, by identifying areas in which the extant theory of middle managers’ role in implementation may need refinement, this review contributes to theory. Finally, findings from this review could inform interventions to leverage middle managers’ role to promote EBP implementation and, subsequently, processes of care and health outcomes.

## Methods

**Fig. 1:**
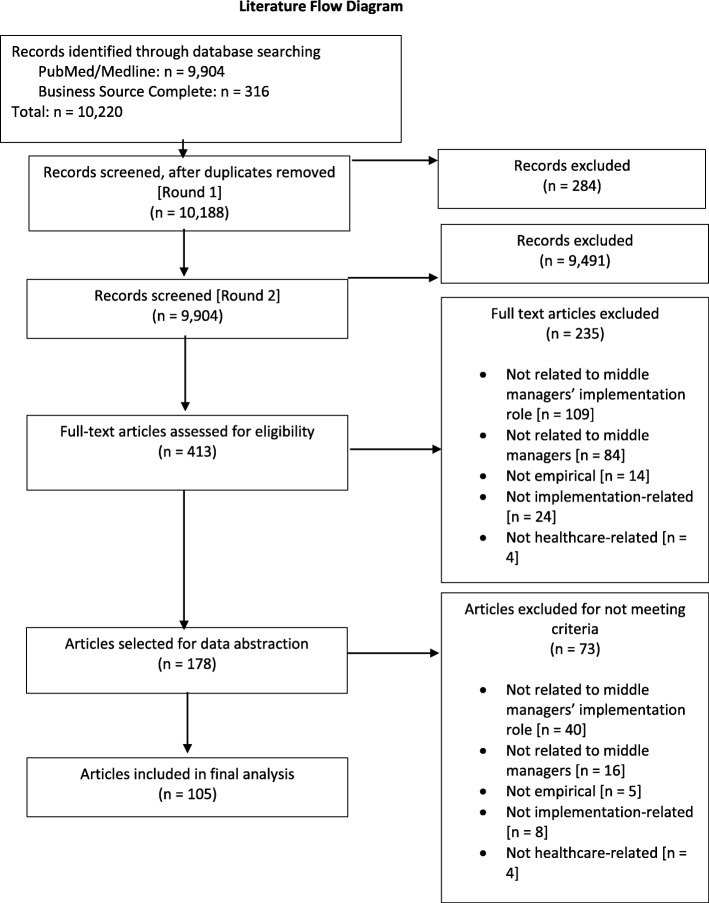
Literature flow diagram

In this study, we performed a systematic search of the literature to identify studies reporting on middle managers’ roles in healthcare EBP implementation and its determinants. We have reported results of the review according to Preferred Reporting Items for Systematic Reviews and Meta-Analyses (PRISMA) guidelines. Figure 1 contains the PRISMA literature flow diagram. The PRISMA checklist is included as Additional file 1.

### Searches

We developed a comprehensive search strategy and conducted the search on January 5, 2015, in MEDLINE/PubMed (1946–2015) and Business Source Complete (Ebsco; 1912–2015). The literature search strategy was developed by the first and last authors (SB and MC) along with a professional medical research librarian (JW) and was intentionally broad so as not to overlook potentially relevant studies. Appropriate subject headings and keywords were identified and categorized into two distinct groups (i) middle manager, leader, supervisor, or other synonym, and (ii) organizational innovation, change, transformation, or other related term (see Additional file 2 for search terms).

### Study inclusion and exclusion criteria

To be included in the study, records were required to relate to middle managers’ role in implementing an EBP and/or its determinants, relate to any area of healthcare (e.g., medical care, mental health, public health, etc.), be written in the English language, peer-reviewed, and report results of an empirical study.

### Study selection process

Pairs of authors independently screened titles and abstracts against the eligibility criteria. Discrepancies were resolved through discussions between members of each pair and, when necessary, the larger research team until consensus was reached. We then screened full-text articles in the same manner.

### Data abstraction and analysis

Given our a priori interest in understanding middle managers’ role in healthcare EBP implementation, we used a modified framework analysis approach [10]. In general, the framework analysis approach allows researchers to analyze data in a matrix format (i.e., Excel workbook) consisting of rows (cases), columns (codes), and cells (summarized data [11]). We adopted a framework analysis approach that included five phases: familiarization, identifying a thematic framework, indexing, charting, and mapping and interpretation. First, in the *familiarization* phase, we reviewed included studies and familiarized ourselves with the literature base. Second, we *identified a thematic framework* based on our specific research objectives to describe studies of middle managers’ role in healthcare EBP implementation and its determinants and the characteristics of these studies. This thematic framework served as the columns (codes) for data abstraction. To describe studies in which researchers have studied middle managers’ role, our thematic framework included objective, setting, phase of implementation (i.e., pre-implementation, during implementation, or post-implementation), design, methods, data sources, and unit of analysis. Consistent with our study objectives, our thematic framework also included middle managers’ role and its determinants. Next, in the *indexing and charting* phases, we abstracted text selections from included articles and placed them into the appropriate cells within our framework. Pairs of authors completed indexing and charting of all included articles. All discrepancies in the indexing and charting phase were discussed until consensus was reached. Finally, in the *mapping and interpretation* phase, we analyzed summarized data from each cell to describe the studies and findings regarding middle managers’ role and its determinants. In many cases, study characteristics were categorical (e.g., observational, quasi-experimental, or experimental design) and summarized using descriptive statistics.

To analyze abstracted data that were qualitative, we used template analysis, using a priori codes and then allowing additional themes to emerge as analysis proceeded. See Additional file 3 for codebooks. We organized EBPs based on a taxonomy proposed by Länsisalmi et al. [12]. We organized findings regarding middle managers’ role based on roles hypothesized by the theory of middle managers’ role in healthcare EBP implementation [1], which builds upon Klein and Sorra’s theory of innovation implementation [9] by expanding its conceptualization of managers’ influence on implementation and has been cited in a growing number of implementation studies [13–15]. We organized findings regarding determinants of middle managers’ role based on the Consolidated Framework for Implementation Research (CFIR), which identifies determinants of implementation primarily at the organizational level, and the Theoretical Domains Framework (TDF), which identifies determinants of implementation primarily at the individual level. In the case of this study, we applied the CFIR constructs first and then used the TDF to expound on any determinants coded as “other individual characteristics,” a construct within CFIR’s “individual characteristics” domain. According to a systematic review on the combined use of CFIR and TDF, supplementing the use of CFIR with the TDF can be a useful approach for more thoroughly conceptualizing determinants at the individual level. We used an inductive approach to code abstracted data regarding the type of middle manager and study objective. Two authors (SB and AAT) coded 20% of study data independently and then met to reconcile coding. The reason for most discrepancies was obvious (e.g., lapse of attention, accidental error).

## Results

The search yielded a total of 10,220 results; 10,188 were screened after duplicates were removed. To ensure inter-rater reliability of reviews, three iterations of sample reviews were conducted with each person reviewing 50 articles (different ones each time) until an average agreement of 83.38% was reached.

Upon title and abstract review, 413 articles were included for full-text review. Based on full-text review, 105 articles were included in our final analysis (Fig. 1). For a full list of included articles, see Additional file 4.

### Description of studies of middle managers’ role in EBP implementation

**Fig. 2:**
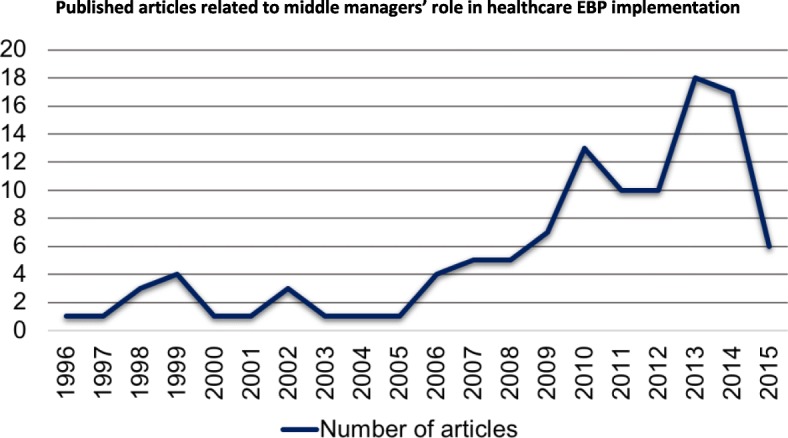
Published articles related to middle managers’ role in healthcare EBP Implementation

As displayed in Fig. 2, the publications on middle managers’ role in healthcare EBP implementation that we identified in our study precipitously increased from 1996 to 2015, with fewer than 5 publications per year through 2008 and 10–20 publications per year thereafter. Table 1 displays study characteristics.

**Table 1:** Study characteristics^a^

Characteristic	*N* (%)
Study objective	
Assess implementation determinants	51 (48.6)
Assess middle managers’ role	13 (12.4)
Assess perceptions of EBP	9 (8.6)
Assess implementation outcomes	9 (8.6)
Assess EBP effectiveness	5 (4.8)
Other	18 (17.1)
Setting	
Hospital/inpatient	39 (37.1)
Outpatient	24 (22.9)
Long-term care facility	9 (8.6)
Multiple	19 (18.1)
Other	14 (13.3)
Country	
USA	38 (36.2)
UK	18 (17.1)
Canada	12 (11.4)
Australia	11 (10.5)
Other	26 (24.8)
EBP	
Regulations, policies, and guidelines	23 (21.9)
Technological innovation (e.g., surgical instrument, MRI)	10 (9.5)
Administrative innovations	16 (15.2)
Operational innovation (e.g., QI initiative to improve lab ordering)	11 (10.5)
Human resources development (e.g., training staff)	10 (9.5)
Multiple (i.e., the study included multiple EBPs)	15 (14.3)
Other	20 (19)
Phase of implementation in which middle managers’ role assessed	
During implementation	32 (30.5)
Post-implementation	19 (18.1)
Pre-implementation	3 (2.9)
Multiple phases	33 (31.4)
Unable to determine	18 (17.1)
Design	
Observational	92 (87.6)
Quasi-experimental	9 (8.6)
Experimental	4 (3.8)
Method	
Qualitative	56 (53.3)
Mixed	41 (39.1)
Quantitative	8 (7.6)
Data collection	
Middle manager as source	
Yes	90 (85.7)
No	9 (8.6)
Unable to determine	6 (5.7)
Unit of data collection	
Individual	76 (72.4)
Organizational	5 (4.8)
Both	24 (22.9)
Source	
Focus groups	1 (1.0)
Observation	2 (1.9)
Survey	4 (3.8)
Interview	32 (30.5)
Multiple	54 (51.4)
Questionnaire	8 (7.6)
Secondary data analysis	4 (3.8)
Questionnaire used^b^	
No	65 (61.9)
Yes	39 (37.1)
Unable to determine	1 (0.9)
Questionnaire validated (*n* = 39)	
No	16 (40)
Yes	10 (25)
Unable to determine	13 (35)
Unit of analysis	
Individual	40 (38.1)
Organizational	38 (36.2)
Both	27 (25.7)

*EBP* evidence-based practice, *QI* quality improvement

^a^*N* = 105 except where otherwise specified

^b^Eight articles used only questionnaires; 31 of the articles used questionnaires as well as other data sources

#### Objective of the study

A plurality (48.6%) of studies’ objectives were to assess determinants of implementation. Studies assessed implementation determinants at various levels, including individuals, organizations, and communities. Thirteen percent of included studies’ objectives focused on middle managers’ role in implementation. Other study objectives included assessing perceptions of interventions (9%); assessing implementation outcomes, including sustainment (9%); and assessing intervention effectiveness (5%).

#### Setting

Thirty-seven percent of studies were conducted in inpatient facilities. Other settings included outpatient (23%), long-term care (8%), and multiple facilities (18%). Most studies were conducted in the USA (36%), UK (18%), or Canada (11%).

#### EBP

A plurality (21.9%) of studies assessed middle managers’ role in implementing regulations, policies, and guidelines. Other EBPs included administrative innovations (15%), operational innovations (10%), technological innovations (e.g., surgical instrument, MRI 9%), or human resources development (e.g., training staff, including middle managers) (9%).

#### Phase of implementation in which middle manager’s role assessed

Studies assessed middle managers’ role across all phases of implementation. Most studies assessed middle managers’ role during implementation (31%) or across multiple phases (31%). We were unable to determine the phase of implementation in 17% of included studies.

#### Design

Eighty-seven percent of included studies were observational, 8% were quasi-experimental, and 4% were experimental. For the quasi-experimental and experimental studies, findings reported on middle managers’ role in implementation were tangential to the overall study objective. As such, these studies did not quantify the relationships of interest for this review (i.e., the relationship between middle managers’ role and implementation, the relationship between determinants and middle managers’ role).

#### Method

Fifty-three percent of included studies were qualitative, 39% were mixed-method, and 8% were quantitative.

#### Data sources

Fifty-one percent of studies used multiple data sources. Thirty percent of studies collected data using interviews, 8% used questionnaires, 4% used secondary organizational data (e.g., administrative data), 4% used surveys, 2% used observational data, and 1% used focus groups.

#### Unit of analysis

Thirty-eight percent of studies analyzed data at the individual level, 36% analyzed at the organizational level, and 26% analyzed at both individual and organizational levels.

Table 2 describes middle managers’ role and determinants’ operationalization and measurement. Most studies (64%) did not define the role of the middle managers who were being assessed a priori.

**Table 2:** Middle managers’ role and determinant operationalization and measurement

Variable	*N* (%)
Middle manager defined	
No	67 (63.8)
Yes	38 (36.2)
Type of middle manager	
Nurse manager	20 (19.0)
Administrative manager (i.e., not clinical)	11 (10.5)
Project manager (i.e., of a time-limited initiative)	39 (37.1)
Unit/team manager (e.g., emergency department manager)	12 (11.4)
Medical director/operations director	10 (9.5)
Multiple	9 (8.6)
Other	4 (3.8)
Middle manager role as outcome	
Yes	91 (86.7)
No	14 (13.3)
Middle manager role actual or desired	
What the role actually was	86 (81.9)
What the role should be	7 (6.7)
Both	4 (3.8)
N/A	8 (7.6)
Article reported middle manager role [as outcome or otherwise]	
Yes	91 (86.7)
No	14 (13.3)
Measure of middle manager role	
Subjective	90 (85.7)
Objective	3 (2.9)
Both	2 (1.9)
Unable to determine	1 (0.9)
N/A (middle managers’ role not measured)	9 (8.6)
How measure was reported if subjective (*n* = 90)	
Self-report	28 (31.1)
Reported by someone else	17 (18.9)
Both	43 (47.8)
Unable to determine	2 (2.2)
Article reported determinant(s) of middle managers’ role	
Yes	45 (42.8)
No	60 (57.2)
Measure of middle manager role determinants	
Subjective	42 (40.0)
Objective	0 (0.0)
Both	0 (0.0)
Unable to determine	2 (1.8)
Not applicable (determinants not measured)	61 (58.1)
How measure was reported if subjective (*n* = 42)	
Self-report	22 (52.4)
Reported by someone else	5 (11.9)
Both	11 (26.2)
Unable to determine	4 (9.5)

*N* = 105 except where otherwise specified

#### Type of middle managers

A plurality of studies (37%) described included middle managers as project managers (e.g., a time-limited project like a quality improvement project, or project supervisor). Nineteen percent of studies included nurse managers, 10% included administrative managers, 9% included medical directors or operations directors, and 11% included team or unit managers. Nine percent of studies included multiple types of middle managers.

### Middle managers’ role

Eighty-seven percent of included studies examined middle managers’ role as an outcome (as opposed to assessing it as a determinant of implementation). Most studies (82%) assessed middle managers’ actual (as opposed to ideal) role (Table 2). Eighty-six percent of included studies assessed middle managers’ role subjectively (i.e., through staff-reported measures), and 2.9% measured it both subjectively and objectively (e.g., through observation or performance data); of these, 48% were based on reports from both middle managers and others, and 31% were based on middle managers’ reports alone. The middle manager roles that included studies identified are displayed in Table 3.

**Table 3:** Middle managers’ roles identified in included studies

Role	*N* (%)^a^	Definition	Exemplar quotes
Mediating between strategy and day-to-day activities	64 (34.6)	Addressing concerns raised by frontline employees and enabling them to fulfill their implementation-related responsibilities by overcoming barriers, holding them accountable, and coaching them.	“Leaders needed to be committed to addressing the administrative, adaptive, and enabling behaviors to find a solution to the adaptive challenge” [28].
Diffusing information	43 (23.2)	Using media to provide comprehensive and relevant information to stakeholders.	“Provide short training courses to keep staff up to date with skills for performance enhancement” [29].
Selling implementation	36 (19.5)	Present, convince and encourage stakeholders to participate in implementation of an innovation.	“Actively provide encouragement and positive feedback” [12].
Synthesizing information	31 (16.8)	Form, combine, interpret, and explain different elements to synthesize a cohesive and comprehensive piece of information.	“Determine QI priorities after analysis of available information such as key performance indicators, patient feedback, and review of organizational strategic directions” [16].
Shaping implementation climate	11 (5.9)	The extent to which EBP implementation is rewarded, supported, and expected in an organization [30]	“Prioritize the implementation of EBP and resource management” [31].

Based on Birken et al. [1, 24] theory of middle managers’ role in innovation implementation. Roles were only counted once per article; some articles included multiple roles

*QI* quality improvement

^a^Ninety-eight studies assessed middle managers’ roles. The statistics displayed reflect the number of roles assessed across the 98 studies

#### Mediating between strategy and day-to-day activities

The most frequently identified role was mediating between strategy and day-to-day activities (34.6%). Mediating between strategy and day-to-day activities involved addressing concerns raised by frontline employees and enabling frontline employees to fulfill their implementation-related responsibilities by overcoming obstacles, holding them accountable, and coaching frontline employees. One middle manager described their role in implementing the Good Goals intervention as follows, “My job is about professional standards... I supervise staff and make sure they are trained, that their workload’s okay, sorting out day-to-day management issues” [16]. Other examples of this role included translating information into concrete tasks that must be executed to implement an EBP, forming strategic staff groups to promote implementation, and measuring employees’ performance related to EBP implementation.

#### Diffusing information

Twenty-three percent of the roles identified involved diffusing information. Diffusing information involved developing comprehensive information, using written and visual media, and providing relevant information to stakeholders. For example, middle managers in one study of the implementation of a quality improvement program used didactic, interactive, and case-based educational sessions for frontline employees [17]. Other examples of middle managers’ role in information diffusion included proactively fielding employees’ questions regarding EBP implementation, providing top managers with feedback regarding status of EBP implementation, and communicating information to external stakeholders.

#### Selling implementation

Nineteen percent of the roles identified involved selling implementation. For example, middle managers in included studies communicated a vision for the EBP and engaged frontline employees in implementation. In a study of nurse leaders implementing a therapeutic nursing approach, middle managers described the benefits of the EBP in staff meetings [18]. In other studies, middle managers’ fulfilled this role by expressing positive attitudes toward EBPs, setting norms related to EBP implementation, and acting as knowledgeable opinion leaders, helping staff to appreciate the rationale for any organizational changes associated with EBP implementation.

#### Synthesizing information

Seventeen percent of the roles identified involved synthesizing information, for example, by defining terms associated with the EBP [1]. In a study of implementing England’s National Health System Change Model, Martin et al. described how middle managers broke the EBP into domains and translated them into language that was “clinician-friendly” [19]. In general, this role involved making more general information about EBP implementation specific to unique organizations and employees, monitoring employee responses to information, and reinterpreting it accordingly to maximize its perceived relevance.

#### Emergent roles

We identified one middle manager role outside of the four roles posited by the theory of middle managers’ role in implementation: shaping implementation climate (6% of identified roles). For example, in one included study of public health agencies implementing quality improvement (QI) initiatives, Davis and colleagues found that middle managers set clear expectations for employee participation in QI by making it part of daily activity [20]. Other examples of ways in which middle managers shaped implementation climate included creating an open, confirming, and evidence-based atmosphere and building an administrative and clinical culture conducive of EBP implementation.

### Determinants of middle managers’ role

Forty-three percent of included studies assessed determinants of middle managers’ role (Table 2). Forty percent of included studies measured determinants of middle managers’ role subjectively; of these, 26% were based on reports from both middle managers and others, and 52% were based on middle managers’ reports alone.

Determinants of middle managers’ role identified in included studies are displayed in Table 4. Table 4 is organized by CFIR domains and constructs. To expound upon the “individual characteristics” domain of CFIR, which Damschroder et al. acknowledged leaving relatively superficial given the availability of frameworks for conceptualizing individual determinants of implementation [21], we conceptualized four TDF constructs: skills, social/professional role and identity, beliefs about capabilities, and competing task demands. CFIR and TDF constructs not included in this table were not found in the data.

**Table 4:** Determinants of middle managers’ roles identified in included studies

Determinant	*N* (%)^a^	Definition	Exemplar quotes
Intervention characteristics			
Evidence strength and quality	6 (5.3)	Middle managers’ perceptions of the quality and validity of evidence supporting the belief that the innovation will have desired outcomes.	“It’s important that ... there’s also some evidence to demonstrate that they [clinicians] are following what is considered best practice” [32].
Outer setting			
External policies and incentives	1 (0.9)	Includes external strategies to spread innovations including policy and regulations (governmental or other central entity), external mandates, recommendations and guidelines, pay-for-performance, collaborative, and public or benchmark reporting.	“Managers from non-adopting districts reported the main reason for not adopting the project were mostly related to the organizational stability as many of them had experienced or anticipated restructurings within the near future” [23].
Inner setting			
Networks and communications	15 (13.2)	The nature and quality of webs of social networks, and the nature and quality of formal and informal communications within an organization	“Inadequate performance measurement due to lack of communication between supervisors and subordinates made setting performance standards challenging” [29].
Implementation climate (leadership engagement)	22 (19.3)	The absorptive capacity for change, shared receptivity of involved individuals to an innovation, and the extent to which use of that innovation will be rewarded, supported, and expected within their organization	“Top managers’ support had a large and significant effect on [middle managers’] commitment; mediators identified were human resources, training, and funding” [4].
Available resources	16 (14.0)	The level of resources organizational dedicated for implementation and on-going operations including physical space and time.	“Implementation of certified practice regulations required the nurse leaders to juggle complex and intersecting fiscal and human resource concerns” [33].
Culture	10 (8.8)	Norms, values, and basic assumptions of a given organization.	“There appeared to be a special synergy at clinics in which administrators and clinicians shared a vision and goals” [34].
Individual characteristics			
Knowledge and beliefs about the EBP	23 (20.2)	Middle managers’ attitudes toward and value placed on the innovation, as well as familiarity with facts, truths, and principles related to the innovation.	“The practice manager’s perception of the ability of the web based care plan to reduce the general practitioner’s workload influenced their decision to encourage the general practitioner to adopt the new system for care planning” [35].
Self-efficacy	1 (0.9)	Middle managers belief in their own capabilities to execute courses of action to achieve implementation goals.	“Many supervisors felt inadequate to supervise EBP implementation” [36].
Other individual characteristics			
TDF: skills	9 (7.9)	Middle managers ability or proficiency acquired through practice.	“Possessing people skills and knowledge of the oncology department workflow was necessary to successfully plan an engaging training” [37].
TDF: beliefs about capabilities	5 (4.4)	Acceptance of the truth, reality or validity about an ability, talent or facility that a person can put to constructive use	“One of the main barriers is fear of exercising authority and being in agencies that do not demand it” [36].
TDF: social/professional role and identity	3 (2.6)	A coherent set of behaviors and displayed personal qualities of an individual in a social or work setting	“One of the key implementation determinants is role/identity (i.e., roles and relationships, working practices, multiple reorganizations, leadership type or lack thereof” [38].
TDF: competing task demands	3 (2.6)	Middle managers conflicting roles and/ or competing demands.	“Some junior managers felt overburdened by their workload” [39].

Determinants were first coded using the Consolidated Framework for Implementation Research [21]. We then used the Theoretical Domains Framework [40] to expound on determinants that were first coded as “other individual characteristics”

*EBP* evidence-based practice

^a^Forty-five studies assessed determinants of middle managers’ roles. The statistics displayed reflect the number of determinants assessed across the 45 studies

#### Inner setting

Nineteen percent of the determinants identified related to the engagement of top managers (i.e., their support and involvement in implementation). Indeed, Balding found that support from top managers increased middle managers’ understanding and ownership of a quality improvement program by allocating resources such as training, time, and incentives [17]. Fourteen percent of the determinants identified related to resources available in a given organization to support EBP implementation (e.g., financial and staffing resources). For example, in their study of residential aged care facilities in Taiwan, Chang et al. found that lack of funding and time negatively influenced nurse managers’ attitudes toward EBP implementation [22]. Other determinants of middle managers’ roles falling within the “inner setting” domain of the CFIR included networks and communications (13%), and culture (9%).

#### Intervention characteristics

Five percent of identified determinants fell within CFIR’s “intervention characteristics” domain. Specifically, these determinants related to the perceived evidence strength and quality of the EBP being implemented.

#### Individual characteristics

Twenty percent of the identified determinants related to middle managers’ knowledge and beliefs about the intervention. For example, Rasmussen et al. found that implementation of an intervention targeting low back pain among elderly care nurses’ aides was less successful when managers did not believe that the intervention would address nurse aides’ needs, reduce nurse aide sick leave, increase nurse aide wellbeing at work, or increase work quality of nurse aides [23]. Other determinants identified in this domain included skills (8%), beliefs about capabilities (4%), social/professional role and identity (3%), competing task demands (3%), engaging key stakeholders (1%), and self-efficacy (1%).

#### Process

One study (1%) assessed engagement of key stakeholders as a determinant of middle managers’ role in implementation.

#### Outer setting

One study (1%) assessed external policies and incentives as a determinant of middle managers’ role in implementation.

## Discussion

The objective of this study was to synthesize extant knowledge of middle managers’ role in healthcare EBP implementation and determinants of those roles, and to reflect on the convergence between extant evidence and the roles hypothesized by the theory of middle managers’ role in implementation. We found that studies on the topic substantially increased over the 1996–2015 period, suggesting an increasing interest in the relationship between middle management and EBP implementation. Studies of middle managers’ role were conducted in several countries and healthcare settings, across multiple implementation phases, and were related to a range of EBPs; this suggests the breadth of contexts in which middle managers may play an important role in implementing innovations and practice changes.

**Fig. 3:**
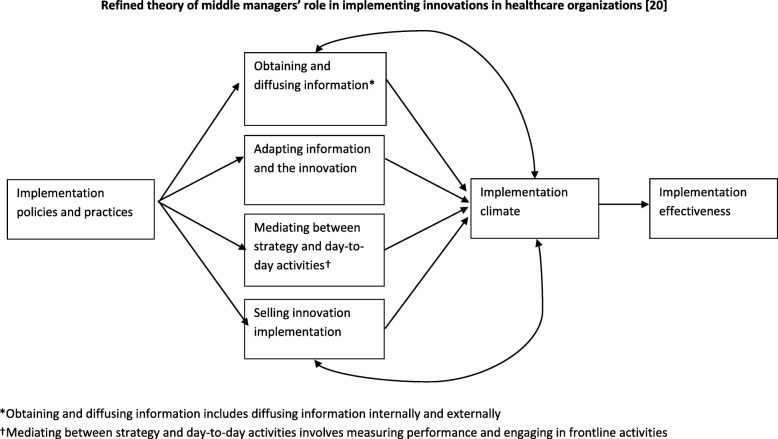
Refined theory of middle managers’ role in implementing innovations in healthcare organizations

We found that most of the middle manager roles that included studies identified were consistent with those hypothesized in the theory of middle managers’ role in implementation; all articles that assessed middle managers’ role found at least one that represented a role hypothesized in this theory [1, 24]. This supports the theory of middle managers’ role in implementation as a useful tool for future research on the topic. In addition to the four roles hypothesized by the theory, included studies found that middle managers help to shape implementation climate. This is consistent with the theory of middle managers’ role in EBP implementation (Fig. 3), which hypothesizes that the relationship between middle managers’ roles and implementation is mediated by implementation climate. That is, the roles that middle managers fulfill in implementation shape implementation climate. Notably, included studies also found that implementation climate influenced middle managers’ roles in implementing EBPs in that the extent to which implementation was rewarded, supported, and expected influenced the roles that middle managers fulfilled in implementing EBPs. Together, these findings suggest a reciprocal relationship between middle managers’ roles and implementation climate, contributing to extant theory of middle managers’ role (see Fig. 3). Future studies should assess whether this reciprocal relationship varies across middle managers’ multiple roles.

Overall, extant studies of middle managers’ role in EBP implementation offer little understanding of determinants of middle managers’ role in EBP implementation. Future studies should seek to elaborate on our understanding of these determinants. Improved understanding of determinants will inform the development of interventions intended to facilitate middle managers’ role in implementation. Some of this work is already underway. For example, Martin et al. [25] leveraged the theory of middle managers’ role to promote clinical supervisors’ engagement in implementing a cognitive behavioral therapy intervention.

Although findings of this review suggest that middle managers assume various roles in EBP implementation—roles that are largely consistent with the extant theory of middle managers’ role in implementation—existing knowledge regarding the relationship between these roles and implementation outcomes is nascent. Understanding of middle managers’ influence on implementation may be enhanced with more experimental and quasi-experimental studies; 88% of included studies were observational, and none of the extant experimental and quasi-experimental studies assessed middle managers’ influence on implementation. Future experimental and quasi-experimental studies should seek to quantify the extent to which different middle manager roles influence implementation outcomes or quantify the influence of determinants on middle managers’ roles or on the relationship between roles and implementation outcomes. This would offer insight on the relative importance of various middle manager roles in implementation and point to levers which could be targeted by future interventions to encourage middle managers to engage in the most important roles. In practice settings, such information could inform the scope of work that middle managers assume in EBP implementation, and suggest tasks, which should be prioritized by middle managers.

Advancing our understanding of middle managers’ role in implementation will also require more systematic approaches to conceptualizing middle managers and their roles in implementation For example, future studies should offer clearer a priori definitions of middle managers, to ensure they can accurately distinguish between top and middle managers. Future studies may also supplement existing subjectively-reported measures of middle managers’ role in implementation with more objective measures of this construct, perhaps through observation. Building and further integrating theory in this area may also help advance our understanding of the topic. For example, Engle et al. expanded on the theory of middle managers’ role in implementation, identifying a set of 14 discrete strategies that middle managers can employ to influence implementation [26]; such a typology could be useful in future studies examining the relative merits of various middle manager actions in facilitating implementation. In practice, a more robust understanding of the relative effectiveness of discrete middle manager actions could inform the job descriptions of middle managers or the training they receive.

Included studies also offered little understanding regarding potential moderators of the relationship between middle managers’ roles and EBP implementation. A plurality of included studies were conducted in inpatient facilities, and the majority were conducted in the USA, the UK, Canada or Australia—notably high-income countries in which middle managers’ roles may systematically differ from lower-income countries; whether middle managers’ influence varies across settings is unclear. Also unclear is whether the relationship depends on the intervention type, implementation phase, or middle manager type. Middle managers may exert more influence on the implementation of changes in delivery systems than clinical practices due to their administrative roles; they may be more influential during implementation than pre-implementation due to the relative importance of mediating between strategy and day-to-day action [24]; and nurse managers may be more influential in implementing EBPs than administrative managers due to their clinical knowledge. Future studies should assess these potential moderators.

Future studies should also assess potential moderators of the influence of these determinants on middle managers’ role. For instance, implementation climate may depend upon the EBP; some EBPs may be more rewarded, supported, and expected than others. The influence of implementation climate on middle managers’ role may depend on senior leadership engagement; more engaged leaders may enhance the influence of implementation climate on middle managers’ role in EBP implementation. Similarly, middle managers’ knowledge and beliefs about EBPs may depend upon the type of middle manager. Nurse managers may have more knowledge about clinical EBPs than administrative managers.

Overall, the results of our systematic review were consistent with a 2015 systematic review of the role and influence of knowledge brokers, who have some similarities to middle managers, in knowledge translation (i.e., implementation [27]). Specifically, the 2015 systematic review found that knowledge brokers fulfilled the role of knowledge manager (i.e., diffusing and synthesizing information) as well as linkage manager and capacity builder (i.e., mediating between strategy and day-to-day action). Also similar to our study, the review found that evidence was insufficient to understand the influence of knowledge brokers on knowledge translation. Our study builds upon the knowledge broker review with understanding of extant evidence regarding middle managers who may have more diverse professional backgrounds, formal roles, and assumed roles in implementation than knowledge brokers.

Several limitations of our study should be considered. To minimize the exclusion of potentially relevant articles, we used very inclusive search terms (see Additional file 2); this approach has several implications. First, there was wide variation in the types of middle managers in studies included in our review, and it is possible that we included some studies related to managers that fall outside many definitions of middle manager. It is also possible that we excluded some relevant studies due to our definition of middle manager. Applying our definition of middle manager in this review was challenging because few studies clearly defined the middle managers that they included. Second, we did not employ a proscriptive definition of “EBP,” but instead considered studies in which a broad range of innovations were implemented. Although this approach allowed us to review a more inclusive sample of potentially relevant literature related to middle managers, it means that innovations implemented in some included studies may not constitute as “evidence-based” by some definitions (e.g., studies in which regulations or policies were implemented). Although we believe that this inclusive approach was necessary to avoid excluding relevant articles, this resulted in more than 10,000 records for title and abstract review. This required the effort of three pairs of reviewers over several months. Consequently, our review excludes articles published after January 2015. Future studies should update this search.

## Conclusions

Despite its limitations, our review offers an initial understanding of extant evidence of middle managers’ roles in EBP implementation. Our findings suggest that middle managers may play a role in facilitating EBP implementation. Further, our findings suggest that there are various determinants of middle managers’ role in EBP implementation. Future research should explore the potential to leverage such determinants to develop interventions to facilitate middle managers’ influence on EBP implementation. Clearer understanding of the moderators of middle managers’ influence on EBP implementation, and its determinants may facilitate the translation of the many EBPs that remain unused in practice.

## Additional files

Additional file 1: PRISMA checklist. (DOC 63 kb)

Additional file 2: Literature search terms. (DOCX 14 kb)

Additional file 3: Codebooks. (DOCX 35 kb)

Additional file 4: Included studies. (DOCX 27 kb)

## Abbreviations

CFIR: Consolidated Framework for Implementation Research
EBPs: Evidence-based practices
QI: Quality improvement
TDF: Theoretical Domains Framework
