# Supplementary material for: Middle managers’ role in implementing evidence-based practices in healthcare: a systematic review
Source: Implement Sci. 2018 Dec 12;13:149. doi: 10.1186/s13012-018-0843-5 (PMC6292008; doi:10.1186/s13012-018-0843-5)
Supplement: Supplementary file 2 — Literature search terms. (DOCX 14 kb) [file 13012_2018_843_MOESM2_ESM.docx]

**Additional file 2**

**Literature Search Terms**

(Organizational innovation [mesh] OR organizational innovation [tiab] OR organisational innovation[tiab] OR innovation implementation[tiab] OR innovative implementation[tiab] OR innovation management[tiab] OR Total Quality Management[tw] OR Lean approach* OR lean principle* OR lean thinking[tw] OR lean method* OR lean management[tw] OR lean manufactur* OR lean healthcare[tw] OR lean health care[tw] OR lean production[tw] OR six sigma[tw] OR Organizational Policy[tw] OR organization transformation[tiab] OR organizational transformation[tiab] OR organisational transformation[tiab] OR organizational change[tiab] OR organisational change[tiab] OR organizational changes[tiab] OR organisational changes[tiab] OR organization change[tiab] OR organisation change[tiab] OR organization changes[tiab] OR organisation changes[tiab] OR Implementation effectiveness[tiab] OR effective implementation[tiab] OR implementation scheme[tiab] OR implementation schemes[tiab] OR strategy implementation[tiab] OR development effectiveness[tiab] OR effective development[tiab] OR adoption[tiab] OR adoption barriers[tiab] OR process improvement[tiab] OR Quality Improvement[tw] OR change management[tiab] OR change process[tiab] OR change processes[tiab] OR Diffusion of innovation[mesh] OR Diffusion of innovation[tiab] OR Diffusing information[tiab] OR Synthesizing information[tiab] OR Evidence-based practice[mh] OR Evidence-based practice[tiab] OR Evidence based practice[tiab] OR Evidence-Based Health Care[tiab] OR Evidence Based Health Care[tiab] OR Evidence-based healthcare[tiab] OR Evidence based healthcare[tiab] OR Evidence-based medicine[tiab] OR Evidence based medicine[tiab] OR Evidence-based nursing[tiab] OR Evidence based nursing[tiab] OR Strategic change[tiab] OR strategic changes[tiab] OR Planned change[tiab] OR planned changes[tiab] OR reengineer* OR re-engineer*) AND ("institutional management teams"[MeSH Terms] OR "management quality circles"[MeSH Terms] OR manager[tiab] OR managers[tiab] OR middle manager[tiab] OR middle managers[tiab] OR middle management[tiab] OR "leadership"[MeSH Terms] OR group leader[tiab] OR group leaders[tiab] OR team leader[tiab] OR team-leader[tiab] OR team leaders[tiab] OR team-leaders[tiab] OR division leaders[tiab] OR leader-follower[tiab] OR leader-member[tiab] OR leader-following[tiab] OR task manager[tiab] OR "nurse administrators"[MeSH Terms] OR nurse administrators[tiab] OR nurse administrator[tiab] OR nursing administrators[tiab] OR nursing administrator[tiab] OR nurse manager[tiab] OR nurse managers[tiab] OR nursing management[tiab] OR nurse leader[tiab] OR nurse leaders[tiab] OR nursing leader[tiab] OR nursing leaders[tiab] OR nurse supervisors[tiab] OR nurse supervisor[tiab] OR physician leader[tiab] OR physician leaders[tiab] OR "hospital administrators"[MeSH Terms] OR hospital administrators[tiab] OR hospital administrator[tiab] OR administrator[tiab] OR program leader[tiab] OR program leaders[tiab] OR director[tiab] OR directors[tiab] OR program director[tiab] OR program directors[tiab] OR (product line management[tiab] OR product line managers[tiab]) OR (service line management[tiab] OR service line manager[tiab] OR service line managers[tiab]) OR knowledge broker[tiab] OR knowledge brokers[tiab] OR knowledge brokerage[tiab] OR boundary spanner[tiab] OR boundary spanners[tiab] OR supervisor[tiab] OR supervisors[tiab] OR "decision making, organizational"[MeSH Terms] OR "knowledge management"[MeSH Terms] OR knowledge management[tiab] OR knowledge manager[tiab])) NOT (case manager[tiab] OR case managed[tiab] OR case managers[tiab] OR case management[tiab]) AND (hasabstract[text] AND English[lang]) NOT (biography[pt] OR personal narratives[pt] OR newspaper article[pt] OR news[pt] OR comment[pt] OR editorial[pt] OR interview[pt] OR letter[pt] OR Portraits[pt] OR Congresses[pt] OR consensus development conference[pt] OR Dictionary[pt] OR lectures[pt] OR legal cases[pt] OR legislation[pt]
