# Supplementary material for: Middle managers’ role in implementing evidence-based practices in healthcare: a systematic review
Source: Implement Sci. 2018 Dec 12;13:149. doi: 10.1186/s13012-018-0843-5 (PMC6292008; doi:10.1186/s13012-018-0843-5)
Supplement: Supplementary file 3 — Codebooks. (DOCX 35 kb) [file 13012_2018_843_MOESM3_ESM.docx]

**Additional File 3**

**Codebooks**

**Middle managers’ role**

NOTES:

1. Coded using constructs from the theory of middle managers’ role in innovation implementation [24].
2. Make sure to code question as well as response when response does not reflect content of question.
3. Code both affirmative and negative responses.
4. Code both roles and influence of roles on implementation climate.

| **Role** | **Definition/Activities** | **Examples** |
| --- | --- | --- |
| Implementation climate | The extent to which EBP implementation is rewarded, supported, and expected in an organization [30] | - Creating a positive climate in the workplace - Building an administrative and clinical culture to align objectives - Create and Support for a culture of evidence-based inter-professional practice - Creating an open, confirming and evidence-based atmosphere. |
| Diffusing information | Using media to provide comprehensive and relevant information to stakeholders. | - Relay information regarding innovation implementation to employees - Stay attuned to top managers’ and frontline employees’ moods and needs - Provide frontline employees with the information necessary to implement innovations - Provide top managers with feedback regarding innovation implementation status - Field employees’ questions regarding innovation implementation - Inform employees of an innovation that is expected to be implemented - Disseminate information regarding material support for innovation implementation - Disseminate information regarding emotional support for innovation implementation - Disseminate rewards for innovation implementation - Obtaining information - Communicating information to external stakeholders |
| Synthesizing information | Form, combine, interpret and explain different elements to synthesize a cohesive and comprehensive piece of information. | - Make general information about innovation implementation relevant to unique organizations and employees - Monitor employees’ responses to the information and reinterpret the information in a way that the employee may find more relevant - Use daily conversations to help frontline employees understand key information regarding innovation implementation - Interpret facts about innovation implementation may convey to employees the relevance of the innovation to the specific roles that they are expected to fulfill - Explain to employees the specific ways in which someone in their role would be supported and rewarded for innovation implementation - Adapting innovation to local context |
| Mediating between strategy and day-to-day activities | Addressing concerns raised by frontline employees and enabling them to fulfill their implementation-related responsibilities by overcoming barriers, holding them accountable, and coaching them. | - Give employees the tools necessary to implement innovations - Translate information into concrete tasks that must be carried out to effectively implement innovations - Provide employees with practical feedback on their innovation implementation-related performance - Form “strategic communities” that promote the implementation of new technologies - Identify specific activities in which employees are expected to engage to promote an organization’s strategy of innovation implementation - Measuring employees’ innovation implementation-related performance - Engaging in innovation implementation-related activities |
| Selling innovation implementation | Present, convince and encourage stakeholders to participate in implementation of an innovation. | - Justify innovation implementation - Knowledgeable opinion leaders who play as a resource for other staff - Encourage employees to consistently and effectively use innovations - Convince employees that innovation implementation is worthy of their attention - Set innovation implementation-related norms - Maintain a positive attitude regarding innovation implementation - Help employees to appreciate the rationale underlying organizational changes |

EBP = evidence-based practice

**Study objective**

1. Coded inductively to identify the codes below.
2. Make sure to code question as well as response when response does not reflect content of question.
3. Code both affirmative and negative responses.

| **Study objective** | **Examples** |
| --- | --- |
| Assess implementation determinants | - Identify factors that may inhibit innovation implementation - Investigate the reasons for poor implementation of an specific policy |
| Assess middle managers’ role | - To strengthen the middle manager role in a hospital QI program - To describes the Clinical Nurse Leader role in implementing person centered are bundles to improve patient outcomes through an innovative culture of caring. - To explore clinical managers’ perceptions of their role in facilitating research utilization and the mechanisms by which they do this |
| Assess perceptions of EBP | - To explore the nurse managers’ perspectives of evidence-based practice - To explore how nurse leaders described and understood their main tasks and roles during a change process. |
| Assess implementation outcomes | - To discuss the knowledge broker model and its implementation, and the broker’s functions and activities, and present the implementation outcome - To understanding how the implementation of the policy influenced outcomes and how was the policy implemented and what factors affected implementation? |
| Assess EBP effectiveness | - Examine the relative effectiveness of a tested smoking cessation education program using two methods of dissemination - Describe the effectiveness of a mentorship program to implement evidence-based practice in a clinical research environment. |

EBP = evidence-based practice

QI = quality improvement

**Evidence-based practice (EBP)**

1. Coded using a taxonomy proposed by Länsisalmi, Kivimäki, Aalto and Ruoranen [11].
2. Make sure to code question as well as response when response does not reflect content of question.
3. Code both affirmative and negative responses.

| **Type of EBP** | **Examples** |
| --- | --- |
| Regulations, policies, and guidelines | - Implement abortion policy - Policy to provide breastfeeding groups for pregnant and breastfeeding women |
| Technological innovation | - Using new surgical instrument, - Utilizing MRI |
| Administrative innovations | - Change Model, a strategy for implementing change in the healthcare setting with the goal to align clinical and administrative needs and practices - Quality improvement efforts to impact medication safety practices and medication error following implementation of electronic medication administration record. |
| Operational innovation | - Use Medical Assessment Units to address quality improvement measures - Improving psychosocial practices in routine mental health care settings |
| Human resources development | - Training of mid- and senior-level healthcare managers - Service improvement projects - Clinical development units have been described as settings committed to developing excellence in nursing practice by introducing the concept of therapeutic nursing, encouraging reflective practice and providing support for nursing research |

**Type of middle manager**

1. Coded inductively to identify the codes below.
2. Make sure to code question as well as response when response does not reflect content of question.
3. Code both affirmative and negative responses.

| **Type of middle manager** | **Definition** | **Examples** |
| --- | --- | --- |
| General manager or supervisor | Healthcare managers generally provide leadership and direction to healthcare organizations, and to divisions, departments, units, or services within those organizations. | - Directorate-level clinical managers - Outpatient substance treatment unit abuse manager - Supervisors of behavioral health therapist - Clinical nursing supervisor |
| Nurse manager | Nurse managers direct, organize and supervise the work of the nursing staff, including registered nurses, licensed practical nurses, certified nursing assistants and medical clerks. | - Nurse manager - Director of nursing - Head nurses - Nurse leaders - Charge nurse - Clinical nurse leader |
| Project leader | Project leaders are responsible for delegating tasks to ensure that staff reach their goals. | - Team leaders - Project leader - Physician leaders - Community mental health team leader |
| Team/unit manager | Unit managers supervise all aspects of operating a unit within a health care organization, from supervising staff to monitoring patient care. | - Unit manager - Unit leader - Unit director - Intensive care unit director - Outpatient substance treatment unit abuse manager |
| Administrative manager | Administrative managers manage support operations by ensuring that information and resources are employed efficiently. | - Administrative manager - Mid-level administrator |
| Director | Directors provide guidance, leadership, oversight and continuous quality improvement for the practice of staff within a predefined area. | - Hospital director - Clinical director - General director - Medical director |
| Department head | Department heads oversee the implementation of long- and short-term strategic plans. | - Heads of institution - Department head - Head of nursing department - Chief clinical officer |

**Determinants of middle managers’ role**

1. Coded using the Consolidated Framework for Implementation Research (CFIR), which identifies determinants of implementation mostly at the organizational level, and the Theoretical Domains Framework (TDF), which identifies determinants of implementation mostly at the individual level. We applied the CFIR constructs first and then used the TDF to expound on any determinants coded as “other individual characteristics.”
2. Make sure to code question as well as response when response does not reflect content of question.
3. Code both affirmative and negative responses.

| **Determinants of middle managers’ role** | **Definition** | **Examples** |
| --- | --- | --- |
| Knowledge and beliefs about the EBP | Middle managers’ attitudes toward and value placed on the innovation, as well as familiarity with facts, truths, and principles related to the innovation. | - Lack of knowledge as the Key barrier to implement EBP - No knowledge of treatment from leadership level as key barrier - Belief that QI improves things for staff & patients |
| Implementation climate (leadership engagement) | The absorptive capacity for change, shared receptivity of involved individuals to an innovation, and the extent to which use of that innovation will be rewarded, supported, and expected within their organization | - Local management was not sufficiently engaged to resolve professional development and career progression issues act as a as key barrier - Manager and commissioner involvement was the most important factor in achieving change |
| Available resources | The level of resources organizational dedicated for implementation and on-going operations including physical space and time. | - Middle managers effectively utilize human resources and funding - Never-ending resource discrepancies between rural programs compared to urban counterparts as key barrier - Lack of training and resources act as key barrier |
| Networks and communications | The nature and quality of webs of social networks, and the nature and quality of formal and informal communications within an organization | - Organizational restructuring exacerbated communication challenges between leaders and with the regulatory board - Experts primarily prefer to utilize verbal - Communication and keep written communication, including e-mail, minimal and brief - Regular communication with peers and supervisors |
| Culture | Norms, values, and basic assumptions of a given organization. | - Systemic barriers included not supportive workplace culture - Small successes, processes and techniques, perseverance, overcoming silo mentality, embracing patient-centered vision, use of data, impact of the innovation |
| Skills | Middle managers ability or proficiency acquired through practice | - Supervisors lack of knowledge about the EBP skills - Possessing people skills and knowledge of each department workflow in order to successfully plan out an engaging training - Belief that they can learn new skills with QI |
| Evidence strength and quality | Middle managers’ perceptions of the quality and validity of evidence supporting the belief that the innovation will have desired outcomes. | - Belief that QI improves things for staff & patients, - Having the perception that QI is a professional obligation, allows for creativity and innovation - The manager's perception of the ability of the web based care plan to reduce the GP's workload |
| Beliefs about capabilities | Acceptance of the truth, reality or validity about an ability, talent or facility that a person can put to constructive use | - Control—of behavior and material and social environment - Perceived competence - Self-confidence/professional confidence - Empowerment Self-esteem - Perceived behavioral control Optimism/pessimism |
| Social/professional role and identity | A coherent set of behaviors and displayed personal qualities of an individual in a social or work setting. | - Professional identity - Professional role - Social identity - Identity - Professional boundaries - Professional confidence - Group identity - Leadership - Organizational commitment - Introduce relational identity as a new sense of self in relation to other staff in other work positions |
| Competing task demands | Middle managers conflicting roles and/ or competing demands. | - Fear of exercising authority and being in agencies that don't demand it - The managers of the non-adopting districts found participating in the project more demanding both regarding economy and time, compared to the adopting districts |
| External policies and incentives | Includes external strategies to spread innovations including policy and regulations (governmental or other central entity), external mandates, recommendations and guidelines, pay-for-performance, collaborative, and public or benchmark reporting. | - Uncertainty in the environment - The need to better understand the financial situation of the organization and the external factors that influence implementation of innovation - Behaviors of the supervisor external to their team such as building relationships with treatment providers within the agency and external stakeholders |
| Self-efficacy | Middle managers belief in their own capabilities to execute courses of action to achieve implementation goals. | - Ability to build the capacity for challenge among staff in different positions." - Ability to acceptance of non-traditional roles in work |

EBP = evidence-based practice

QI = quality improvement
