# Supplementary material for: Middle managers’ role in implementing evidence-based practices in healthcare: a systematic review
Source: Implement Sci. 2018 Dec 12;13:149. doi: 10.1186/s13012-018-0843-5 (PMC6292008; doi:10.1186/s13012-018-0843-5)
Supplement: Supplementary file 4 — Included studies. (DOCX 27 kb) [file 13012_2018_843_MOESM4_ESM.docx]

**Additional file 4**

**Included Studies**

1. Aagaard EM, Gonzales R, Camargo Jr CA, et al. Physician champions are key to improving antibiotic prescribing quality. *The Joint Commission Journal on Quality and Patient Safety.* 2010;36(3):109-116.

2. Adaji A, Schattner P, Piterman L. Web based diabetes care planning: Sociotechnical barriers to implementation in general practice. *Australian family physician.* 2011;40(11):915.

3. Amodeo M, Storti SA, Larson MJ. Moving empirically supported practices to addiction treatment programs: recruiting supervisors to help in technology transfer. *Substance use & misuse.* 2010;45(6):968-982.

4. Aniteye P, Mayhew SH. Shaping legal abortion provision in Ghana: using policy theory to understand provider-related obstacles to policy implementation. *Health Research Policy and Systems.* 2013;11(1):23.

5. Atsalos C, O'brien L, Jackson D. Against the odds: experiences of nurse leaders in Clinical Development Units (Nursing) in Australia. *Journal of advanced nursing.* 2007;58(6):576-584.

6. Balding C. Embedding organisational quality improvement through middle manager ownership. *International Journal of Health Care Quality Assurance.* 2005;18(4):271-288.

7. Berkhout AJ, Boumans NP, Mur I, Nijhuis FJ. Conditions for successfully implementing resident‐oriented care in nursing homes. *Scandinavian journal of caring sciences.* 2009;23(2):298-308.

8. Birken SA, Lee S-YD, Weiner BJ, Chin MH, Chiu M, Schaefer CT. From strategy to action: How top managers’ support increases middle managers’ commitment to innovation implementation in healthcare organizations. *Health care management review.* 2015;40(2):159.

9. Birken SA, Lee S-YD, Weiner BJ, Chin MH, Schaefer CT. Improving the effectiveness of health care innovation implementation: middle managers as change agents. *Medical Care Research and Review.* 2013;70(1):29-45.

10. Boorsma M, Langedijk E, Frijters DH, Nijpels G, Elfring T, van Hout HP. Implementation of geriatric assessment and decision support in residential care homes: facilitating and impeding factors during initial and maintenance phase. *BMC health services research.* 2013;13(1):8.

11. Boström AM, Wallin L, Nordström G. Evidence‐based practice and determinants of research use in elderly care in Sweden. *Journal of evaluation in clinical practice.* 2007;13(4):665-673.

12. Boumans N, Landeweerd J. A Dutch study of the effects of primary nursing on job characteristics and organizational processes. *Journal of Advanced Nursing.* 1996;24(1):16-23.

13. Boyson C, Taylor S, Page L. The National Heatwave Plan–A Brief Evaluation of Issues for Frontline Health Staff. *PLoS currents.* 2014;6.

14. Brooks H, Pilgrim D, Rogers A. Innovation in mental health services: what are the key components of success? *Implementation Science.* 2011;6(1):120.

15. Bungay V, Stevenson J. Nurse Leaders’ Experiences of Implementing Regulatory Changes in Sexual Health Nursing Practice in British Columbia, Canada. *Policy, Politics, & Nursing Practice.* 2013;14(2):69-78.

16. Caine C, Kenrick M. The role of clinical directorate managers in facilitating evidence‐based practice: a report of an exploratory study. *Journal of Nursing Management.* 1997;5(3):157-165.

17. Caldwell DF, Chatman J, O'Reilly III CA, Ormiston M, Lapiz M. Implementing strategic change in a health care system: The importance of leadership and change readiness. *Health care management review.* 2008;33(2):124-133.

18. Carlson L, Rapp CA, Eichler MS. The experts rate: Supervisory behaviors that impact the implementation of evidence-based practices. *Community Mental Health Journal.* 2012;48(2):179-186.

19. Champagne F, Lemieux-Charles L, Duranceau M-F, MacKean G, Reay T. Organizational impact of evidence-informed decision making training initiatives: a case study comparison of two approaches. *Implementation Science.* 2014;9(1):53.

20. Chandhiok N, Joglekar N, Shrotri A, Choudhury P, Chaudhury N, Singh S. Task-shifting challenges for provision of skilled birth attendance: a qualitative exploration. *International health.* 2014;7(3):195-203.

21. Chang HC, Jones MK, Russell C. Exploring attitudes and barriers toward the use of evidence-based nursing among nurse managers in Taiwanese residential aged care facilities. *Journal of gerontological nursing.* 2013;39(2):36-42.

22. Clapp JD. Organizational factors related to AIDS/HIV education in outpatient substance abuse treatment units. *Journal of health & social policy.* 1998;9(3):1-13.

23. Cooke M, Mattick R, Campbell E. A description of the adoption of the'Fresh start'smoking cessation program by antenatal clinic managers. *The Australian journal of advanced nursing: a quarterly publication of the Royal Australian Nursing Federation.* 2000;18(1):13-21.

24. Cooke M, Mattick RP, Campbell E. The dissemination of a smoking cessation program to 23 antenatal clinics: the predictors of initial program adoption by managers. *Australian and New Zealand journal of public health.* 1999;23(1):99-103.

25. Daiker BL. Adaptive challenges in medical practices. *Journal of Medical Practice Management.* 2013;28:332-340.

26. Döpp CM, Graff MJ, Rikkert MGO, van der Sanden MWN, Vernooij-Dassen MJ. Determinants for the effectiveness of implementing an occupational therapy intervention in routine dementia care. *Implementation science.* 2013;8(1):131.

27. Douglas NF, Hinckley JJ, Haley WE, Andel R, Chisolm TH, Eddins AC. Perceptions of speech-language pathologists linked to evidence-based practice use in skilled nursing facilities. *American journal of speech-language pathology.* 2014;23(4):612-624.

28. Eggenberger T, Garrison H, Hilton N, Giovengo K. Discharge phone calls: using person‐centred communication to improve outcomes. *Journal of nursing management.* 2013;21(5):733-739.

29. Etheridge F, Tannenbaum C, Couturier Y. A systemwide formula for continence care: overcoming barriers, clarifying solutions, and defining team members’ roles. *Journal of the American Medical Directors Association.* 2008;9(3):178-189.

30. Fletcher J, Gavin M, Harkness E, Gask L. A collaborative approach to embedding graduate primary care mental health workers in the UK National Health Service. *Health & social care in the community.* 2008;16(5):451-459.

31. Fontaine P, Whitebird R, Solberg LI, Tillema J, Smithson A, Crabtree BF. Minnesota’s early experience with medical home implementation: viewpoints from the front lines. *Journal of general internal medicine.* 2015;30(7):899-906.

32. Gagliardi A, Majewski C, Victor J, Baker G. Quality improvement capacity: a survey of hospital quality managers. *BMJ Quality & Safety.* 2010;19(1):27-30.

33. Gifford W, Davies B, Edwards N, Graham I. Leadership strategies to influence the use of clinical practice guidelines. *Nursing Leadership (Toronto, Ont).* 2006;19(4):72-88.

34. Gilson L, Elloker S, Olckers P, Lehmann U. Advancing the application of systems thinking in health: South African examples of a leadership of sensemaking for primary health care. *Health Research Policy and Systems.* 2014;12(1):30.

35. Girard A, Rochette A, Fillion B. Knowledge translation and improving practices in neurological rehabilitation: managers' viewpoint. *Journal of evaluation in clinical practice.* 2013;19(1):60-67.

36. Gowen III CR, Henagan SC, McFadden KL. Knowledge management as a mediator for the efficacy of transformational leadership and quality management initiatives in US health care. *Health care management review.* 2009;34(2):129-140.

37. Guerrero EG. Managerial capacity and adoption of culturally competent practices in outpatient substance abuse treatment organizations. *Journal of Substance Abuse Treatment.* 2010;39(4):329-339.

38. Hailey D, Yu P, Munyisia EN. Pre-implementation investigation of the readiness of allied health professionals to adopt electronic health records. Paper presented at: HIC2014.

39. Hall C, Sigford B, Sayer N. Practice Changes Associated with the Department of Veterans Affairs′ Family Care Collaborative. *Journal of general internal medicine.* 2010;25(1):18-26.

40. Harvey G, Jas P, Walshe K. Analysing organisational context: case studies on the contribution of absorptive capacity theory to understanding inter-organisational variation in performance improvement. *BMJ Qual Saf.* 2014:bmjqs-2014-002928.

41. Henggeler SW, Schoenwald SK, Liao JG, Letourneau EJ, Edwards DL. Transporting efficacious treatments to field settings: The link between supervisory practices and therapist fidelity in MST programs. *Journal of Clinical Child and Adolescent Psychology.* 2002;31(2):155-167.

42. Hewison A. Nurse managers’ narratives of organizational change in the English National Health Service. *Journal of nursing management.* 2012;20(7):858-867.

43. Hoagwood KE, Kelleher K, Murray LK, Jensen PS. Implementation of evidence-based practices for children in four countries: a project of the World Psychiatric Association. *Revista Brasileira de Psiquiatria.* 2006;28(1):59-66.

44. Hoddinott P, Britten J, Pill R. Why do interventions work in some places and not others: a breastfeeding support group trial. *Social Science & Medicine.* 2010;70(5):769-778.

45. Hopkins JR. Financial incentives for ambulatory care performance improvement. *The Joint Commission journal on quality improvement.* 1999;25(5):223-238.

46. Hugelius K, Berg S, Westerberg E, Gifford M, Adolfsson A. Swedish ambulance managers’ descriptions of crisis support for ambulance staff after potentially traumatic events. *Prehospital and disaster medicine.* 2014;29(6):589-592.

47. Huijg JM, van der Zouwe N, Crone MR, Verheijden MW, Middelkoop BJ, Gebhardt WA. Factors influencing the introduction of physical activity interventions in primary health care: a qualitative study. *International journal of behavioral medicine.* 2015;22(3):404-414.

48. Inamdar SN, Osland A, Wells P. Restructuring with the middle-management advantage. *The health care manager.* 2010;29(4):305-317.

49. Ingersoll GL, Cook J-A, Fogel S, Applegate M, Frank B. The effect of patient-focused redesign on midlevel nurse managers' role responsibilities and work environment. *Journal of Nursing Administration.* 1999;29(5):21-27.

50. Janols R, Lind T, Göransson B, Sandblad B. Evaluation of user adoption during three module deployments of region-wide electronic patient record systems. *International journal of medical informatics.* 2014;83(6):438-449.

51. Jeffs LP, Lo J, Beswick S, Campbell H. Implementing an organization-wide quality improvement initiative: insights from project leads, managers, and frontline nurses. *Nursing administration quarterly.* 2013;37(3):222-230.

52. Kahn JM, Matthews FA, Angus DC, Barnato AE, Rubenfeld GD. Barriers to implementing the Leapfrog Group recommendations for intensivist physician staffing: a survey of intensive care unit directors. *Journal of critical care.* 2007;22(2):97-103.

53. Kellogg KC. Operating room: Relational spaces and microinstitutional change in surgery. *American Journal of Sociology.* 2009;115(3):657-711.

54. Kertesz SG, Austin EL, Holmes SK, et al. Making housing first happen: organizational leadership in VA’s expansion of permanent supportive housing. *Journal of general internal medicine.* 2014;29(4):835-844.

55. Khresheh R, Barclay L. Implementation of a new birth record in three hospitals in Jordan: a study of health system improvement. *Health Policy and Planning.* 2007;23(1):76-82.

56. Kitson A, Silverston H, Wiechula R, Zeitz K, Marcoionni D, Page T. Clinical nursing leaders’, team members’ and service managers’ experiences of implementing evidence at a local level. *Journal of Nursing Management.* 2011;19(4):542-555.

57. Klingner J, Moscovice I, Tupper J, Coburn A, Wakefield M. Implementing patient safety initiatives in rural hospitals. *The Journal of Rural Health.* 2009;25(4):352-357.

58. Knight AW, Caesar C, Ford D, Coughlin A, Frick C. Improving primary care in Australia through the Australian Primary Care Collaboratives Program: a quality improvement report. *BMJ Qual saf.* 2012:bmjqs-2011-000165.

59. Knox AB, Underbaake G, McBride PE, Mejicano GC. Organization development strategies for continuing medical education. *Journal of Continuing Education in the Health Professions.* 2001;21(1):15-23.

60. Kolehmainen N, MacLennan G, Ternent L, et al. Using shared goal setting to improve access and equity: a mixed methods study of the Good Goals intervention in children’s occupational therapy. *Implementation Science.* 2012;7(1):76.

61. Lunkka N, Suhonen M. Ambiguous meanings of projects as facilitators of sensegiving. *Journal of nursing management.* 2015;23(7):842-850.

62. Lutwama GW, Roos JH, Dolamo BL. Assessing the implementation of performance management of health care workers in Uganda. *BMC health services research.* 2013;13(1):355.

63. Mackintosh N, Watson K, Rance S, Sandall J. Value of a modified early obstetric warning system (MEOWS) in managing maternal complications in the peripartum period: an ethnographic study. *BMJ Qual Saf.* 2013:bmjqs-2012-001781.

64. Macphee M, Suryaprakash N. First‐line nurse leaders’ health‐care change management initiatives. *Journal of Nursing Management.* 2012;20(2):249-259.

65. Marshall MN. Improving quality in general practice: qualitative case study of barriers faced by health authorities. *Bmj.* 1999;319(7203):164-167.

66. Martin GP, Sutton E, Willars J, Dixon-Woods M. Frameworks for change in healthcare organisations: A formative evaluation of the NHS Change Model. *Health services management research.* 2013;26(2-3):65-75.

67. Mash B, Mayers P, Conradie H, Orayn A, Kuiper M, Marais J. How to manage organisational change and create practice teams: experiences of a South African primary care health centre. *Education for Health.* 2008;21(2):132.

68. McMullen CK, Schneider J, Firemark A, Davis J, Spofford M. Cultivating engaged leadership through a learning collaborative: lessons from primary care renewal in Oregon safety net clinics. *The Annals of Family Medicine.* 2013;11(Suppl 1):S34-S40.

69. Mohammad Mosadeghrad A. Why TQM does not work in Iranian healthcare organisations. *International journal of health care quality assurance.* 2014;27(4):320-335.

70. Morrow E, Robert G, Maben J. Exploring the nature and impact of leadership on the local implementation of The Productive Ward Releasing Time to Care™. *Journal of health organization and management.* 2014;28(2):154-176.

71. Munoz-Plaza CE, Strauss SM, Astone-Twerwll JM, Des Jarlais DC, Hagan H. Staff perspectives on facilitating the implementation of hepatitis C services at drug treatment programs. *Journal of psychoactive drugs.* 2006;38(3):233-241.

72. Newman M, Papadopoulos I, Sigsworth J. Barriers to evidence-based practice. *Intensive and Critical Care Nursing.* 1998;14(5):231-238.

73. Ogden T, Bjørnebekk G, Kjøbli J, et al. Measurement of implementation components ten years after a nationwide introduction of empirically supported programs–a pilot study. *Implementation Science.* 2012;7(1):49.

74. Parker LE, Kirchner JE, Bonner LM, et al. Creating a quality-improvement dialogue: utilizing knowledge from frontline staff, managers, and experts to foster health care quality improvement. *Qualitative health research.* 2009;19(2):229-242.

75. Parsons ML, Cornett PA. Sustaining the pivotal organizational outcome: magnet recognition. *Journal of Nursing Management.* 2011;19(2):277-286.

76. Pogoda TK, Cramer IE, Rosenheck RA, Resnick SG. Qualitative analysis of barriers to implementation of supported employment in the Department of Veterans Affairs. *Psychiatric Services.* 2011;62(11):1289-1295.

77. Ramsay AI, Turner S, Cavell G, et al. Governing patient safety: lessons learned from a mixed methods evaluation of implementing a ward-level medication safety scorecard in two English NHS hospitals. *BMJ Qual Saf.* 2013:bmjqs-2012-001730.

78. Rantz MJ, Zwygart-Stauffacher M, Flesner M, et al. Challenges of using quality improvement methods in nursing homes that “need improvement”. *Journal of the American Medical Directors Association.* 2012;13(8):732-738.

79. Rantz MJ, Zwygart-Stauffacher M, Flesner M, et al. The influence of teams to sustain quality improvement in nursing homes that “need improvement”. *Journal of the American Medical Directors Association.* 2013;14(1):48-52.

80. Rapp CA, Etzel-Wise D, Marty D, et al. Barriers to evidence-based practice implementation: Results of a qualitative study. *Community Mental Health Journal.* 2010;46(2):112-118.

81. Rasmussen CDN, Larsen AK, Holtermann A, Søgaard K, Jørgensen MB. Adoption of workplaces and reach of employees for a multi-faceted intervention targeting low back pain among nurses’ aides. *BMC medical research methodology.* 2014;14(1):60.

82. Reay T, Golden‐Biddle K, Germann K. Challenges and leadership strategies for managers of nurse practitioners. *Journal of nursing management.* 2003;11(6):396-403.

83. Rees G, Huby G, McDade L, McKechnie L. Joint working in community mental health teams: implementation of an integrated care pathway. *Health & social care in the community.* 2004;12(6):527-536.

84. Saint S, Kowalski CP, Banaszak-Holl J, Forman J, Damschroder L, Krein SL. How active resisters and organizational constipators affect health care–acquired infection prevention efforts. *The Joint Commission Journal on Quality and Patient Safety.* 2009;35(5):239-246.

85. Saint S, Kowalski CP, Banaszak-Holl J, Forman J, Damschroder L, Krein SL. The importance of leadership in preventing healthcare-associated infection: results of a multisite qualitative study. *Infection Control & Hospital Epidemiology.* 2010;31(9):901-907.

86. Salmela S, Eriksson K, Fagerström L. Leading change: a three‐dimensional model of nurse leaders’ main tasks and roles during a change process. *Journal of advanced nursing.* 2012;68(2):423-433.

87. Sinuff T, Cook D, Giacomini M, Heyland D, Dodek P. Facilitating clinician adherence to guidelines in the intensive care unit: a multicenter, qualitative study. *Critical care medicine.* 2007;35(9):2083-2089.

88. Sorensen R, Paull G, Magann L, Davis J. Managing between the agendas: implementing health care reform policy in an acute care hospital. *Journal of health organization and management.* 2013;27(6):698-713.

89. Spyridonidis D, Calnan M. Opening the black box: a study of the process of NICE guidelines implementation. *Health Policy.* 2011;102(2-3):117-125.

90. Stelfox HT, Straus SE, Nathens A, Gruen RL, Hameed SM, Kirkpatrick A. Trauma center quality improvement programs in the United States, Canada, and Australasia. *Annals of surgery.* 2012;256(1):163-169.

91. Stetler CB, Ritchie JA, Rycroft‐Malone J, Charns MP. Leadership for evidence‐based practice: strategic and functional behaviors for institutionalizing EBP. *Worldviews on Evidence‐Based Nursing.* 2014;11(4):219-226.

92. Sunaert P, Bastiaens H, Feyen L, et al. Implementation of a program for type 2 diabetes based on the Chronic Care Model in a hospital-centered health care system:" the Belgian experience". *BMC Health Services Research.* 2009;9(1):152.

93. Suter E, Arndt J, Lait J, et al. How can frontline managers demonstrate leadership in enabling interprofessional practice? Paper presented at: Healthcare management forum2007.

94. Swain K, Whitley R, McHugo GJ, Drake RE. The sustainability of evidence-based practices in routine mental health agencies. *Community Mental Health Journal.* 2010;46(2):119-129.

95. Turner T, Short J, Group SOS. Barriers to and enablers of evidence‐based practice in perinatal care in the SEA‐ORCHID project. *Journal of evaluation in clinical practice.* 2013;19(4):591-597.

96. Urquhart R, Porter GA, Grunfeld E. Reflections on knowledge brokering within a multidisciplinary research team. *Journal of Continuing Education in the Health Professions.* 2011;31(4):283-290.

97. van Hamersveld KT, den Bakker E, Nyamtema AS, et al. Barriers to conducting effective obstetric audit in Ifakara: a qualitative assessment in an under‐resourced setting in Tanzania. *Tropical Medicine & International Health.* 2012;17(5):652-657.

98. Vogelsmeier A, Scott-Cawiezell J. Achieving quality improvement in the nursing home: influence of nursing leadership on communication and teamwork. *Journal of nursing care quality.* 2011;26(3):236-242.

99. Wallen GR, Mitchell SA, Melnyk B, et al. Implementing evidence‐based practice: effectiveness of a structured multifaceted mentorship programme. *Journal of advanced nursing.* 2010;66(12):2761-2771.

100. Walley P, Silvester K, Mountford S. Health-care process improvement decisions: a systems perspective. *International Journal of Health Care Quality Assurance.* 2006;19(1):93-104.

101. Wilkinson JE, Nutley SM, Davies HT. An exploration of the roles of nurse managers in evidence‐based practice implementation. *Worldviews on Evidence‐Based Nursing.* 2011;8(4):236-246.

102. Williams R, Woodell C, McCarville E, et al. Desired attributes and skills of program managers in translation of evidence-based interventions. *Health promotion practice.* 2011;12(6_suppl_1):82S-90S.

103. Willmot M. The new ward manager: an evaluation of the changing role of the charge nurse. *Journal of advanced nursing.* 1998;28(2):419-427.

104. Yeatman H, Nove T. Reorienting health services with capacity building: a case study of the Core Skills in Health Promotion Project. *Health Promotion International.* 2002;17(4):341-350.

105. Yu P, Gandhidasan S, Miller AA. Different usage of the same oncology information system in two hospitals in Sydney—Lessons go beyond the initial introduction. *International Journal of Medical Informatics.* 2010;79(6):422-429.
